# Exploring the Thalamus as a Target for Neuropathic Pain Management: An Integrative Review

**DOI:** 10.7759/cureus.60130

**Published:** 2024-05-12

**Authors:** Mariana P Pires, Billy McBenedict, Issra E Ahmed, Ryan Chun Chien Yau, Yan Bin Fong, Kang Suen Goh, Yee Siew Lim, Suber Abdi Mohamed, Owen Ngu, Jeshua N Devan, Wilhelmina N Hauwanga, Bruno Lima Pessôa

**Affiliations:** 1 Neurosurgery, Fluminense Federal University, Niterói, BRA; 2 Surgery, National Ribat University, Khartoum, SDN; 3 Internal Medicine, Monash University Malaysia, Subang Jaya, MYS; 4 Surgery, Universiti Putra Malaysia, Serdang, MYS; 5 Surgery, International Medical University, Seremban, MYS; 6 Medicine, Jiangsu University, Zhenjiang Jiangbin Hospital, Zhenjiang, CHN; 7 Medicine, University of Malaya, Kuala Lumpur, MYS; 8 Surgery, Asian Institute of Medicine, Science and Technology University, Bedong, MYS; 9 Family Medicine, Faculty of Medicine, Federal University of the State of Rio de Janeiro, Rio de Janeiro, BRA

**Keywords:** pain network, thalamotomy, thalamus, neuropathic pain, dbs

## Abstract

Neuropathic pain (NP), resulting from damage to the somatosensory system, is characterized by either spontaneous or evoked pain. In the context of NP, wherein aberrant signaling pathways contribute to the perception of pain, the thalamus emerges as a key player. This structure is integral to the pain network that includes connections to the dorsal horn of the spinal cord, highlighting its role in the affective-motivational aspects of pain perception. Given its significant involvement, the thalamus is targeted in advanced treatments such as thalamotomy and deep brain stimulation (DBS) when traditional therapies fail, emphasizing the need to understand its function in NP to improve management strategies. This review aimed to provide an overview of the role of the thalamus in the transmission of nociceptive information in NP by discussing the existing evidence, including the effectiveness and safety of current techniques in the management and treatment of NP. This is an integrative review involving the qualitative analysis of scientific articles published in PubMed/MEDLINE, Embase, Scopus, and Web of Science. A total of 687 articles were identified, and after selection, 15 articles were included in this study. All studies reviewed demonstrated varying degrees of effectiveness of DBS and thalamotomy in alleviating painful symptoms, although the relief was often temporary. Many studies noted a reduction in pain perception at the conclusion of treatment compared to pre-treatment levels, with this decrease maintained throughout patient follow-ups. However, adverse events associated with these treatments were also reported. In conclusion, there are some benefits, albeit temporary, to using thalamotomy and DBS to alleviate the painful symptoms of NP. Both procedures are considered advanced forms of surgical intervention that aim to modulate pain pathways in the brain, providing significant relief for patients suffering from chronic pain resistant to conventional treatment. Despite limitations, these surgical interventions offer renewed hope for patients facing disabling chronic pain and can provide a significant improvement in quality of life.

## Introduction and background

Neuropathic pain (NP) can be spontaneous or evoked and is the result of an injury or disease affecting the somatosensory system [1]. Globally, the prevalence of NP is estimated to be approximately 5%-10% of the population [2]. Furthermore, affected patients demonstrate greater spontaneous activity in the ventral posterior thalamus compared to patients who suffer from movement disorders but do not experience NP. This connection between the prevalence of NP and neuronal activity in the thalamus suggests a relationship between the condition and the neural response in the brain, highlighting the complexity and importance of understanding NP, its clinical manifestations, and its relationship to the thalamus [3]. The thalamus is located in the diencephalon, close to the center of the brain, performing numerous functions and establishing intricate connections with different regions within the brain. Thalamocortical neurons have a dual function, acting not only as conduits for the transmission of nociceptive information but also as participants in its processing before sending it to the cerebral cortex [4]. Considering its complex connections and its role in sensory processing, the thalamus plays a fundamental role in the perception and regulation of pain signals. The dual nociceptive pathway demonstrates that this structure receives nociceptive signals, which are responsible for transmitting sensations of pain. These signals travel through the spinal cord until they reach the thalamus [5].

The thalamus plays an indispensable role in modulating ascending nociceptive input and is an essential component of a network that projects to the dorsal horn of the spinal cord [6]. This dynamic involvement further demonstrates its importance in regulating the affective-motivational components of pain perception. Studies describe that in patients with chronic NP, the thalamus exhibits increased levels of burst firing [7]. From a functional imaging perspective, it has been demonstrated that NP is fundamentally different from non-neuropathic pain and can be altered or generated by structural changes in the thalamus [8]. There is increasing evidence of a critical role of the thalamus in the generation and/or persistence of NP. For example, NP is associated with altered thalamic anatomy [9], changes in thalamic biochemistry and decreased thalamic perfusion [10], and thalamocortical dysrhythmia [11]. Over the last four decades, the thalamus has been the target structure for many ablative surgeries, and when conventional treatments become ineffective, thalamotomy becomes a treatment option to be considered. Another option to consider is deep brain stimulation (DBS) for patients with NP [12]. This further reinforces the importance of understanding this structure, as well as its relationship with NP, which could greatly benefit patients in managing this type of pain in the future. Thus, this integrative review aimed to provide an overview of the role of the thalamus in the transmission of nociceptive information in NP by discussing the existing evidence, including the effectiveness and safety of current techniques in the management and treatment of NP.

## Review

Materials and methods

Search Strategy

We conducted a comprehensive search across multiple databases, including PubMed/MEDLINE, Embase, Scopus, and Web of Science. Our search utilized relevant keywords and medical subject heading (MeSH) terms to identify articles related to the role of the thalamus in managing NP. The following keywords were transformed into PubMed MeSH terms for efficient retrieval: “Thalamus,” “Thalamic,” “Neuropathic pain,” “Neuralgia,” “Chronic pain,” and “Management.” We employed Boolean operators (“OR” and “AND”) to create a consistent search across these databases, and Table 1 provides the details regarding the search strategy. This review utilized the Preferred Reporting Items for Systematic Reviews and Meta-Analyses (PRISMA) guidelines.

**Table 1:** Summary of the search strategy from the databases

Database	Search Strategy	Filters Used
PubMed/MEDLINE	("thalamus" OR "thalamic") AND ("neuropathic pain" OR "neuralgia" OR "chronic pain") AND ("management" OR "treatment")	Humans only, English language, exclude preprints, filter years 2012-2024
Embase	(thalamus:ab,ti OR thalamic:ab,ti) AND ('neuropathic pain':ab,ti OR 'neuralgia':ab,ti OR 'chronic pain':ab,ti) AND (management:ab,ti OR treatment:ab,ti)	Humans only, English language, exclude preprints, filter years 2012-2024
Scopus	("thalamus" OR "thalamic") AND ( "neuropathic pain" OR "neuralgia" OR "chronic pain" ) AND ( "management" OR "treatment")	Humans only, English language, exclude preprints, filter years 2012-2024
Web of Science	("thalamus" OR "thalamic") (Abstract) AND ("neuropathic pain" OR "neuralgia" OR "chronic pain") (Abstract) AND ("management" OR "treatment") (Abstract)	Humans only, English language, exclude preprints, filter years 2012-2024

Study Eligibility Criteria

Our search targeted online records in English and focused exclusively on studies involving human participants. We included a range of study designs, such as randomized controlled trials, prospective and retrospective studies, clinical trials, and observational studies. These studies were selected based on their contributions to understanding the role of the thalamus in chronic pain. Acceptable materials consisted solely of primary research and reviews published in peer-reviewed journals. We excluded non-human or animal studies, in vitro studies not directly relevant to the thalamus and chronic pain, non-peer-reviewed articles, conference abstracts, editorials, and any duplicate publications or redundant data from the same research group. Based on the results obtained, two independent reviewers analyzed the title and abstract of the studies found to assess their appropriateness for the research objective.

Results

**Figure 1:**
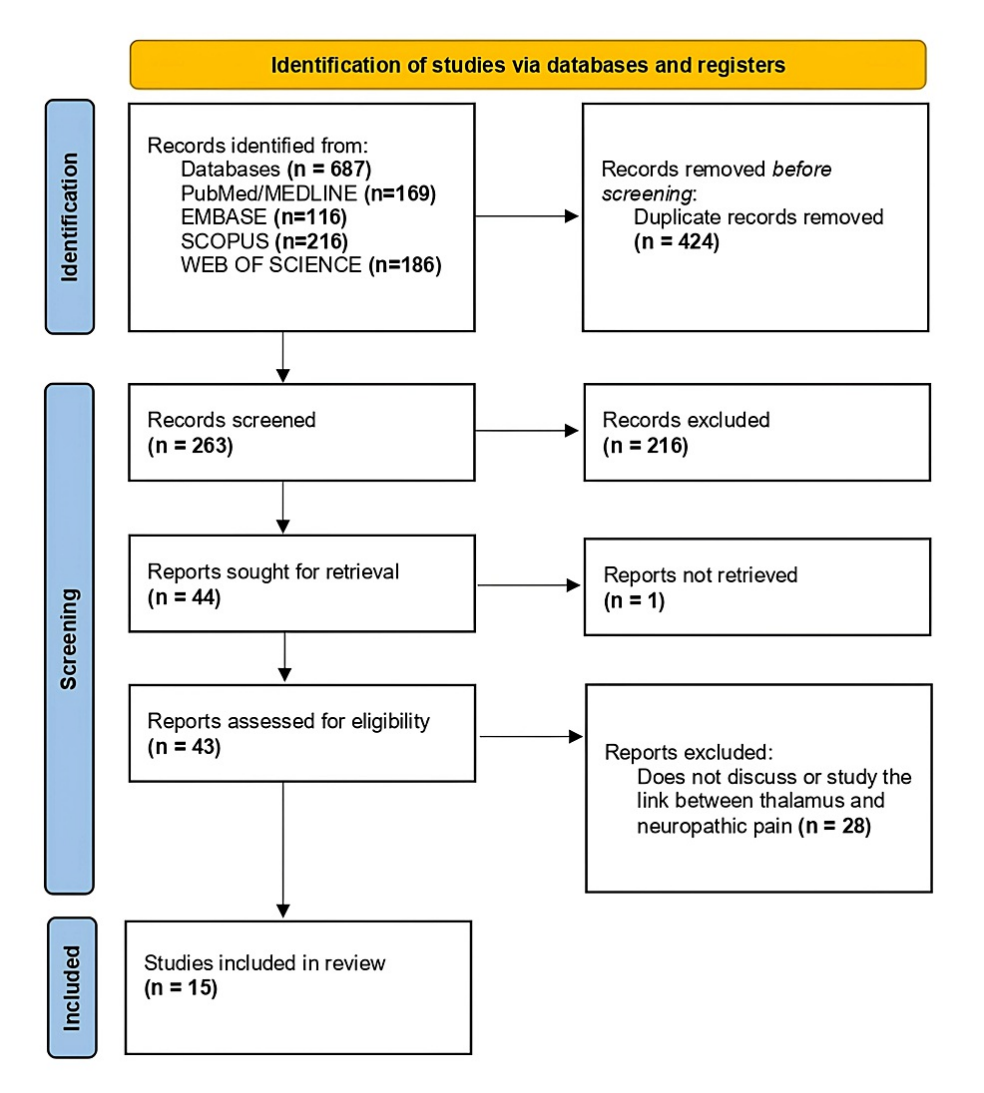
Flow diagram of the Preferred Reporting Items for Systematic Reviews and Meta-Analyses (PRISMA), illustrating the article selection process for this review

Through our search strategy, we identified a total of 687 articles, which included (1) 169 articles from PubMed and MEDLINE; (2) 116 articles through Embase; (3) 216 articles on Scopus; and (4) 186 articles from Web of Science (Figure 1). The articles were imported into Zotero software, where duplicates were removed, leaving us with 263 records, and these were further reduced to 43 after exclusion based on the abstracts. The 43 articles were read in full and assessed for eligibility according to our inclusion/exclusion criteria, leaving 15 articles that met the criteria.

The 43 articles selected were thoroughly reviewed to extract information pertinent to the study. Of these, only 15 met our inclusion criteria. The key findings from these 15 articles were systematically summarized (Table 2). The data compiled included details such as the authors, number of participants, and specific causes of pain addressed in each study. This structured approach facilitated a clear presentation of the diverse research findings related to our investigation.

**Table 2:** Studies that were used to synthesize this review, with their respective key results

Authors	Participants	Cause of Pain	Key Findings
Alshelh et al. [13]	58 patients (44 healthy controls)	Modified thalamic firing and thalamocortical dysrhythmia	In subjects with neuropathic pain (NP), a heightened astrocyte oscillatory activity within the dorsal horn and/or somatosensory thalamus caused an amplification of thalamocortical oscillatory activity. This showed a self-sustaining thalamocortical dysrhythmia, resulting in persistent pain perception.
Danyluk et al. [14]	43 patients (20 healthy controls)	Medically refractory trigeminal neuralgia	Multiple abnormalities in thalamic structure and metabolism are observed in patients with medically refractory trigeminal neuralgia undergoing surgery. Those who respond inadequately to surgery demonstrate baseline disparities in thalamic shape and experience varying trajectories of early postoperative changes in thalamic metabolism compared to responders.
Gustin et al. [15]	43 patients (21 healthy controls)	Orofacial NP	NP following spinal cord injury is related to a significant decrease in thalamic N-acetyl aspartate and gamma-aminobutyric acid content. Blood circulation in the area surrounding the thalamic reticular nucleus decreases as well.
Abreu et al. [16]	16 patients	Chronic NP due to traumatic injuries	During the follow-up in the third year, deep brain stimulation (DBS) is shown to be effective for chronic NP following traumatic amputation and brachial plexus injury. The reduction in pain persisted across all pain assessment measures, with patients with phantom limb pain having improved slightly more.
Gallay et al. [17]	55 patients	Intractable chronic NP	After 55 months of mean follow-up period, participants experienced an average pain reduction of 42%, with over 50% of them experiencing a minimum 50% alleviation in pain. Participants with classical and idiopathic trigeminal neuralgia were observed to have a more substantial average pain reduction compared to the total sample.
Son et al. [18]	9 patients	Spinal cord injury (SCI) pain, arm amputation stump pain, central post-stroke pain (CPSP)	A reduction in pain of 37.9% ± 16.5% was noted in the chronic motor cortex stimulation (MCS) group (n=6), while the chronic DBS group (n=2) had a decrease in pain of 37.5%. No statistically significant difference in pain reduction level comparing participants with MCS and DBS.
Krüger et al. [19]	1 patient	Dental NP	The centromedian, ventral posterior medial, and anterior pulvinar were the three potential nuclei that can be targeted with deep brain stimulation in managing neuropathic dental pain. Optimal outcomes were observed during ventral posterior medial (VPM) stimulation, resulting in more than 90% decrease in pain, and centromedian (CM) stimulation, leading to a 50% decrease in pain. After three months of VPM-DBS combined with lateral CM stimulation, the participants had a significant decrease in their pain disability index (from 25 to 0) and improvement in their short form 36 scores (from 67.5 to 90).
Urgosik and Liscak [20]	30 patients	Severe treatment-resistant pain syndromes	43.3% had successful treatments of pain relief, and 56.7% of patients had very little to no pain relief. The pain reduction duration was 10–72 months.
Pirrotta et al. [21]	8 patients	Thalamus pain syndrome and other causes of pain associated with somatosensory systems	Pain relief noted in patients after central lateral thalamotomy (CLT). Significant improvement in patients’ depression scores three months after CLT.
Lovo et al. [22]	10 patients	Trigeminal neuralgia and complex pain syndromes	60% success rate according to Barrow Neurological Institute Pain Scale of I to IIIb. 50% of the patients had 50% of pain reduction during final follow-up. 75% of patients with trigeminal neuralgia responded to the treatment.
Taranta et al. [23]	66 patients	Somatosensory system lesions or disease	Magnetic resonance-guided focused ultrasound targeting the thalamus showed to be safe and effective, representing a new potential treatment for medically refractory NP. Follow-up after one year showed the mean Mini-Mental State Examination score (29.4 ± 1.3; p = 0.2) as well as the mean Montreal Cognitive Assessment score (28.0 ± 2.8; p = 0.02) had improved.
Danyluk et al. [24]	22 patients	Trigeminal neuralgia	The length of disease demonstrated a negative correlation to the connectivity between the anterior cingulate cortex (ACC) and the left amygdala (r^2^ = 0.34, p = 0.00437) as well as with the right hippocampus (r^2^ = 0.21, p = 0.0318).
Lovo et al. [25]	10 patients	Compression of the trigeminal nerve	Two (25%) patients had no pain visual analogue scale (VAS 0), three (37.5%) had mild pain (VAS 1 to 3), and three (37.5%) had moderate pain (VAS 4 to 7) ​​24 hours after treatment. Forty-eight hours after treatment, all patients reported pain relief, seven (87.5%) reported more than 50%, and one (12.5%) patient reported 30% relief.
Kim et al. [26]	256 patients	Lesions or diseases involving the somatosensory system	Among the 62 functional magnetic resonance imaging studies, there is a significant reduction in the activity of primary somatosensory and motor cortex (SI and MI), thalamus, insula, and ACC following treatments compared to baseline. Across the 13 PET studies, glucose uptake, blood flow, and opioid-receptor binding potentials had a significant increment in SI, MI, thalamus, and insula post-treatment compared to baseline.
Gray et al. [27]	18 patients	Phantom limb pain, post-stroke pain, cephalalgia	Significant improvements were noted by using the McGill Pain Questionnaire, with it decreases from 31.94 before surgery to 18.44 after surgery.

Discussion

Anatomy of the Thalamus

**Figure 2:**
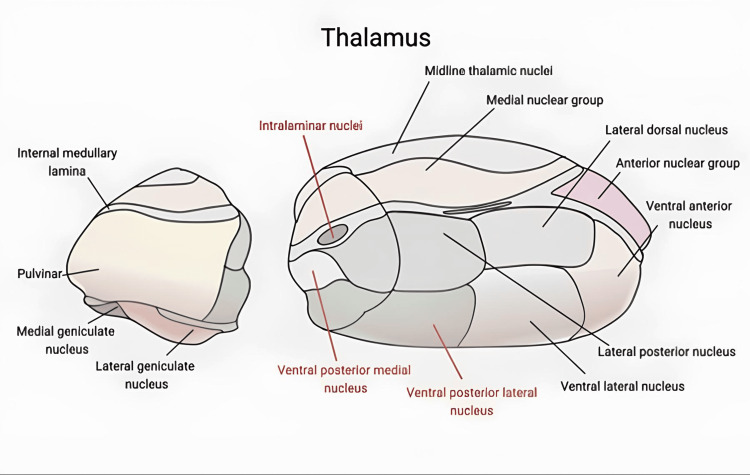
Illustration of the thalamus, highlighting the intralaminar nuclei, ventral posterior medial nucleus, and ventral posterior lateral nucleus Created by the authors using biorender.com

The thalamus, located centrally in the brain (Figure 2), plays a crucial role in transmitting facial pain through its involvement in the trigeminal sensory pathway [22]. It is divided into several distinct regions, including the anterior nucleus, the ventral lateral nucleus, and the ventral posterior nucleus, which is further subdivided into the ventral posterior lateral (VPL) and ventral posterior medial (VPM) nuclei. Other important areas include the lateral geniculate nucleus, the medial geniculate nucleus, and the pulvinar nucleus [14,22]. Medial structures such as the centromedian (CM) nucleus of the thalamus and parafascicular (Pfc) complexes serve as relay nuclei in the interpretation of pain and are targeted by medial thalamotomy in the treatment of neurogenic and chronic deafferentation pain typically resulting from neurological damage [22]. Additionally, the interlaminar, dorsomedial, and CM regions are key relay areas for the paleospinothalamic tracts and are associated with chronic pain management, as they transmit sensitive and affective information [22].

Related to sensorimotor functions, the central lateral (CL) nuclei and the posterior part of the CL nucleus (CLp) are integral to the salience and attention networks. These nuclei receive nociceptive information from the spinothalamic and spino-reticulo-thalamic pathways, which are then transmitted to cortical areas. The processing of somatosensory and nociceptive information primarily involves the VPM and VPL nuclei. These nuclei also play a crucial role in deeper cognitive functions that influence the matrix and perception of pain. Additionally, the sensory-discriminatory system is impacted by cells from the spinothalamic tract projecting to the lateral thalamus, highlighting another vital function of these nuclei [23]. An anatomical aspect of relevance is the observation by Danyluk et al. [24], who noted that the volume of the thalamus contralateral to the side of pain was enlarged in patients with trigeminal neuralgia (TN), suggesting structural changes associated with chronic pain conditions [14].

The Role of the Thalamus in Pain

The thalamus serves as a crucial hub for transmitting acute noxious signals from peripheral receptors to the cortex via the spinothalamic pathway. Although the physiological mechanisms underlying the development and maintenance of NP are not fully understood, some human studies suggest that this condition is linked with altered thalamic burst firing and thalamocortical dysrhythmia. A study observed that individuals with orofacial NP exhibited increased infraslow oscillatory activity along the ascending pain pathway, including the spinal trigeminal nucleus, somatosensory thalamus, thalamic reticular nucleus (TRN), and primary somatosensory cortex [13]. These findings support the hypothesis that oscillatory activity in the ascending pain pathway may contribute to enhanced thalamocortical oscillatory activity, persistent thalamocortical dysrhythmia, and frequent pain perception [13]. Thalamic pain syndrome, a type of centralized pain, stems from damage to the central nervous system, particularly the thalamus, often following a cerebrovascular accident such as a stroke. This NP is characterized by hyperalgesia and allodynia, where patients become hypersensitive due to central sensitization, a state where the nervous system remains in high activity, enhancing neural signaling and reducing the threshold to fire action potentials. This phenomenon, known clinically as temporal summation or "wind-up," results in severe sensory deficits, including changes in temperature perception. Thalamic pain can be triggered by various types of strokes, including ischemic and hemorrhagic, with lesions such as a lacunar infarct or a more extensive infarction like that of the middle cerebral artery. Additionally, the pain can present acutely, subacutely, or even years post-injury, complicating the management and treatment of this debilitating condition [28].

Although the precise mechanism of the analgesic effect of thalamic DBS remains unknown, it is theorized to involve the activation of thalamocortical pathways and alterations in cortical activity [18]. The CL nucleus has significant cortical projections that reach extensive cortical domains, positioning it to relay nociceptive information received via the spinothalamic and spino-reticulo-thalamic tracts to areas involved in nociception. It also receives known afferents from the spinothalamic tract. Additionally, in patients with chronic pain, the left posterior medial thalamus shows reduced activation during pain-related tasks after medical treatment compared to before treatment, suggesting an effect of the treatment. This observation was supported by a multiple comparison-corrected activation likelihood estimation (ALE) analysis, indicating that the medial posterior thalamus is notably impacted by pain in these patients [13]. Some studies found that the thalamus was activated by pain tasks in both patients and healthy controls, but the control group exhibited significantly stronger thalamic activity during pain experiences [26,28-30]. Additionally, an analysis reported that patients with TN demonstrated increased preoperative functional connectivity between the right insular cortex and both the left and right thalamus. Conversely, those who did not respond to surgery showed greater functional connectivity among limbic structures, including between the bilateral hippocampus. Disease duration was also observed to negatively affect connectivity between the anterior cingulate cortex and the left amygdala and right hippocampus [24].

Regarding the modulatory effects on pain, stimulation of the thalamus has been shown to influence cerebral blood flow in the insula, as evidenced by studies [13,31-33]. Changes in the anatomy and biochemistry of the thalamus, including decreased thalamic perfusion, may be associated with the development of NP. There is increasing evidence suggesting that NP is linked to thalamocortical dysrhythmia, a condition that may also affect the inhibitory action of the TRN. The decreased perfusion in the TRN region, observed in individuals with orofacial NP, could be associated with reduced levels of gamma amino butyric acid (GABA) in the thalamus [15]. Elevated levels of glutamatergic neurotransmitters may result from heightened activity in primary afferent neurons or lesions in the nervous system, potentially leading to neuronal death. This increase is often accompanied by the loss of GABAergic interneurons in the dorsal horn, a phenomenon observed in animal models of NP [34,35]. Additionally, excessive neural discharge can trigger astrocyte activation, leading to various changes such as increased infraslow neural oscillations and calcium waves along the ascending pain pathway. In the somatosensory thalamus, local reductions in interneurons contribute to decreased activity in the TRN and reduced GABAergic output [36]. These alterations in thalamocortical connectivity perpetuate the continuous perception of pain and enhance the dominance of the ascending pain pathway, ultimately magnifying the centrality of this pathway in the process of pain modulation.

Effectiveness of Thalamotomy and DBS in NP Management

Based on the hypothesis concerning the role of the medial thalamic center in the pathophysiology of pain, medial thalamotomies began to be performed at the end of the 1940s. By 1990, through multiarchitectonic studies and intraoperative single-cell recordings, this area was confirmed as a surgical target, leading to the development of functional radiosurgical procedures in this region [20]. Additionally, in the late 1940s, functional neurosurgeons conducted medial thalamotomies to treat NP [17]. Recent results from Gamma Knife surgery have been promising in patients with tremors, and the results from transcranial magnetic resonance-guided focused ultrasound (MRgFUS) thalamotomy have also been encouraging in patients with treatment-refractory pain [20]. The anterior cingulate gyrus and the thalamus are the primary anatomical sites for DBS in the treatment of NP. Historically, the practice of thalamic DBS has not been widespread, primarily due to misconceptions about its efficacy. Initially, DBS was misunderstood because of the partial, insufficient, or only short-term effectiveness of cortical stimulation when first introduced [37]. However, the effectiveness of thalamic DBS has significantly improved, and many studies now demonstrate its efficacy and relatively fewer side effects. In the context of NP, the effectiveness of DBS is generally defined as achieving more than 50% pain improvement, with results ranging from 40% to 60% in published studies. More recently, the anterior and dorsal cingulate gyrus have been proposed as new targets for DBS in cases of refractory pain [38].

In a study involving 25 patients undergoing thalamic DBS for the treatment of chronic NP and 18 patients with motor cortex stimulation (MCS) over the past 10 years, nine patients underwent a simultaneous trial of DBS and MCS. Patients refractory to medical and pharmacological treatments were referred for surgery. Their causes of pain were central post-stroke pain, spinal cord injury pain, thoracolumbar injury, transition zone pain, iatrogenic spinal cord transection, central cervical syrinx pain, and pain in the arm amputation stump. In patients with a minimum visual analogue scale (VAS) score of 7/10, a simultaneous trial of MCS and DBS was considered. Initially implanted with both DBS and MCS electrodes, eight out of nine patients (89%) had a successful trial; 75% of these eight patients responded to MCS, and the remaining two responded to Vc DBS [18]. Thalamotomy, particularly in the posterior part of the central lateral (CLp) nucleus, is justified by the alteration or loss of normal function in this area over time, which contributes to the sustainment and amplification of deleterious low-frequency activity, one of the causes of thalamocortical dysrhythmia. This dysrhythmic state is characterized by increased low- and high-frequency cortical activities, manifesting within the pain matrix. Quantitative EEG recordings have confirmed that this dysrhythmic state sustains and amplifies harmful low-frequency signals. The efficacy of targeting the CLp for thalamotomy has been demonstrated by Gallay et al. [17], who reported a 31.4% pain relief with stereotactic central lateral thalamotomy (CLT). This finding is further supported in other studies [39,40], where an average pain reduction of 53% in a larger patient cohort was observed. These results highlight the effectiveness of targeting the CLp in alleviating pain associated with thalamocortical dysrhythmia [21].

In the treatment of NP, thalamotomy, especially bilateral medial thalamotomies centered on the CLp nucleus, has been shown to be more effective than unilateral contralateral thalamotomies for managing drug-refractory NP. This effectiveness aligns with findings from prior research, which revealed that the presence of NP correlates with increases in both the low- and high-frequency spectra of the spectral electroencephalogram [23]. Furthermore, cells from the spinothalamic tract project to the area of the CLp nucleus in the medial thalamus and collateralize to the lateral thalamus, further supporting the efficacy of targeting this specific area in thalamotomy procedures [23]. For over 50 years, DBS has been utilized to ameliorate chronic pain and is a well-established treatment for Parkinson's disease, dystonia, and other movement disorders. It is also used off-label for conditions such as epilepsy, obsessive-compulsive and psychiatric disorders, Tourette syndrome, and cluster headaches. A study reported the outcome of contralateral ventroposterolateral sensory thalamic DBS in 16 patients with chronic NP over a period of 29 months [16]. In this study, one patient with a brachial plexus injury did not respond to the postoperative DBS trial. However, the remaining 15 patients proceeded with implantation. Despite this, the average pain relief was sustained even after 36 months, with the median and interquartile range of VAS score improvement recorded at 52.8% (45.4%) (p = 0.00021). Notably, there were no surgical complications or side effects associated with the stimulation. Thus, DBS demonstrated a three-year efficacy in treating chronic NP resulting from traumatic amputation and brachial plexus injury [16].

With regard to thalamotomy, the decision to use an ablative procedure to treat functional disorders has been disregarded by some professionals. Although overlooked, in recent years, there has been a renaissance in wound interventions for the treatment of various clinical conditions [20]. The performance of a CL nucleus thalamotomy is based on the observation that this region has lost its normal function over time (less than 1% of cells with receptive fields and absence of deficits after CLT). It appears to sustain or amplify a deleterious overproduction of low frequency, approximately 4 Hz, which is the source of a dysfunctional thalamocortical mechanism known as thalamocortical dysrhythmia [17]. A non-randomized, single-center, retrospective cross-sectional analysis evaluated 112 CLT targets in patients suffering from chronic, therapy-resistant NP [17]. CLT was administered bilaterally to 48 patients, contralaterally to seven patients relative to the pain, and through repeated MRgFUS interventions in eight patients. The study documented only one serious adverse event, a case of numbness in the upper lip, with an average follow-up duration of 55 months. Notably, pain relief of at least 30% was reported by 65% of patients at three months, 63% at one year, and 61% at the final follow-up. Furthermore, VAS scores decreased by 41% for continuous pain relief and 49% for pain attacks at the one-year follow-up. These findings demonstrate that MRgFUS CLT is a stable and safe treatment option for NP, providing significant pain relief for a substantial proportion of patients [17].

Studies With Positive Results of Thalamotomy and DBS

Recent studies have explored innovative approaches to alleviate pain, particularly focusing on the stimulation of the VPM nucleus of the thalamus. This technique specifically targets neural pathways involved in pain perception. VPM stimulation has demonstrated remarkable efficacy in reducing pain, achieving an impressive 90% decrease in pain intensity [19]. However, it is important to note that these findings are based on a singular case of success documented in the existing literature. The VPM nucleus, which is critical in the physiological pain pathway, receives secondary trigeminal afferents from the head, face, and oral structures via the trigeminal lemniscus of the caudal nucleus. This specific nucleus has also been a focal point in other studies investigating DBS for chronic facial pain conditions. Notably, 10 out of 13 individuals treated with VPM DBS for chronic facial pain reported significant benefits from this technique [19].

Previous research has demonstrated that stimulation effectively reduces subjective pain intensity. According to a study [27], improvements were observed in the McGill Pain Questionnaire (MPQ) and the Bodily Pain subscale of the SF-36, with a mean pain reduction of 44.7% using the MPQ. This reduction surpasses the 30% threshold recommended by the Initiative on Methods, Measurement, and Pain Assessment in Clinical Trials for significant improvement in patient-reported pain outcomes. Notably, 39% of participants reported a pain decrease of more than 50%. Additionally, stimulation had a positive impact on quality of life, as evidenced by significant improvements in physical and mental role limitations. In contrast, another study noted changes in general health and social functioning post-surgery [27], which may be influenced by the specific types of pain experienced by participants. Although statistical analysis comparing the quality of life improvements across different pain groups was limited by the sample size, it is worth noting that changes in the general health subscale approached statistical significance, with a p-value of 0.054 [27].

In the study conducted by Gallay et al. [17], the focus was on assessing pain relief outcomes after CLT, which was performed bilaterally on 112 targets across 48 patients. Additionally, repeated interventions using MRgFUS were carried out. Three months post-procedure, patients reported an average pain reduction of 42% ± 32%, which marginally increased to 43% ± 36% after one year. At the final follow-up, which had an average duration of 55 months, the patient-rated relief stabilized at 42% ± 37% (n = 63). Notably, 65% of patients achieved at least a 30% reduction in pain at the three-month mark, and 54% reported favorable outcomes, defined as at least 50% pain relief. The mean VAS scores mirrored these reductions in pain. Impressively, there was a 92% decrease in the average number of pain attacks, and allodynia was mitigated or eliminated in 68% of the patients post-MRgFUS CLT. The study highlights that many patients achieved long-term pain relief, with some reporting more than a 50% reduction in pain even two years after the procedure [17].

Indications and Controversies Regarding Thalamotomy and DBS

Thalamotomy and DBS are neurosurgical interventions used to manage complex neurological conditions. Specifically, DBS targeting the periventricular gray and periaqueductal gray areas has been indicated as a treatment option for patients with intractable chronic pain syndromes, particularly when other treatment modalities have failed to provide relief [18]. The European Federation of Neurological Societies and the UK National Institute for Health and Clinical Excellence endorse DBS as a treatment for NP. However, despite its approval for some neurological conditions, the use of DBS for NP remains "off-label" in some countries. This is partly because two multicenter trials conducted to obtain US FDA approval did not meet the efficacy criteria, which required at least half of the patients to achieve at least 50% pain relief one year post-surgery [16].

Traditional DBS targeting solely the thalamus has encountered challenges, especially in treating central pain syndromes, where a multi-target approach might be more effective. In one study, researchers focused on patients suffering from severe painful attacks of TN. When percutaneous procedures failed to alleviate symptoms, stereotactic radiosurgery (SRS) was considered as an alternative. The innovative approach involved simultaneous treatment of the affected trigeminal nerve and the contralateral CM and posterior commissure using SRS. This strategy aimed to achieve rapid pain relief within 72 hours by targeting multiple areas beyond just the pituitary gland. The results were promising, providing clinical proof of concept that a multi-target SRS approach can effectively alleviate pain in suitable patients [25].

The MRgFUS CLT has been used for patients who have not seen improvement from antiepileptic and antidepressant medications over at least one year [17]. This approach addresses the ineffectiveness or adverse effects of conventional treatments, highlighting the ongoing search for more effective and less invasive therapeutic options for refractory neurological conditions [17]. Gallay et al.'s [17] retrospective study of 63 interventions illustrates a challenge: the desired outcomes often take time to manifest. Specifically, in this study, pain reduction was more significant at the one-year follow-up compared to the initial three months post-surgery, demonstrating the gradual effectiveness of MRgFUS CLT in managing chronic and therapy-resistant NP [17].

## Conclusions

The thalamus plays a pivotal role in the transmission and modulation of NP, serving as a crucial hub within the neural pathways that convey noxious signals to the cortex. Research highlights its involvement in altered neural dynamics, such as thalamic burst firing and thalamocortical dysrhythmia, which are associated with the chronicity and intensity of pain. Thalamic pain syndrome underscores the central role of thalamic damage in NP, which is characterized by persistent and challenging-to-treat symptoms. Moreover, interventions such as DBS and thalamotomy, targeting specific thalamic regions, have shown potential for managing NP, although the exact mechanisms remain to be fully elucidated. These findings emphasize the complexity of pain perception and the central role of the thalamus in its modulation, suggesting that targeted thalamic interventions could enhance therapeutic outcomes for patients suffering from NP.
